# Adjunctive photodynamic therapy improves outcomes and quality of life in condyloma acuminatum after radical cervical cancer surgery

**DOI:** 10.3389/fonc.2026.1848211

**Published:** 2026-06-23

**Authors:** Cheng Zhang, Wei Zheng, Xiuli Wang, Zihan Wang

**Affiliations:** 1 Gynecology Department, Yantai Yuhuangding Hospital, Yantai, China; 2 Dermatology Department, Yantai Mountain Hospital, Yantai, China

**Keywords:** cervical cancer, CO2 laser, condyloma acuminatum, photodynamic therapy, quality of life

## Abstract

**Background:**

Condyloma acuminatum (CA), caused by human papillomavirus infection, frequently persists or recurs in patients undergoing treatment for cervical cancer and can significantly impair postoperative quality of life. Purpose: This study aimed to compare the efficacy of photodynamic therapy (PDT) combined with CO_2_ laser versus CO_2_ laser monotherapy in alleviating condyloma acuminatum (CA) symptoms and improving quality of life in patients with early-stage cervical cancer complicated by CA after radical surgery.

**Methods:**

In this retrospective study, 148 patients were divided into a single laser group (n=81) receiving only CO_2_ laser and a combined PDT group (n=67) receiving both CO_2_ laser and PDT. Primary outcomes included CA symptom relief (assessed at 6 months post-treatment) and quality of life evaluated using the Dermatology Life Quality Index (DLQI), Female Sexual Function Index (FSFI), and European Organization for Research and Treatment of Cancer Quality of Life Questionnaire-Core 30 (EORTC QLQ-C30) at baseline and 6 months. Secondary outcomes comprised human papillomavirus (HPV), a viral load, and serum cytokine levels measured at baseline and 4 weeks, as well as recorded adverse reactions.

**Results:**

The combined PDT group demonstrated a significantly higher overall symptom relief rate (94.03% vs. 77.78%, P = 0.006) and lower recurrence rates at 3 months (7.46% vs. 24.69%) and 6 months (11.94% vs. 34.57%) compared to the single laser group. Greater improvements were observed in DLQI (10.46 vs. 11.73, P = 0.001), FSFI scores (25.09 vs. 23.83, P = 0.013), and across multiple EORTC QLQ-C30 domains—including physical, role, emotional, and social functioning, as well as global health status (all P<0.05) in the combined group. Viral load reduction was more pronounced (0.97 vs. 2.44 ×10^6^ copies/mL, P<0.001), accompanied by a more favorable cytokine profile shift. Adverse reactions differed, with more erythema/edema but less scarring in the combined PDT group (all P<0.05).

**Conclusion:**

The addition of photodynamic therapy, a light-based immunomodulatory approach within photomedicine, enhances clinical outcomes and supports improved quality of life, potentially through synergistic cytotoxic and immune-mediated mechanisms.

## 1 Introduction

Condyloma acuminatum (CA), a prevalent manifestation of low-risk human papillomavirus (HPV) infection, represents a common and clinically significant comorbidity in patients diagnosed with early-stage cervical cancer (1, 2). The management of these patients extends beyond oncologic surgery, as the presence of symptomatic CA can significantly impair physical comfort, psychological well-being, and quality of life during the critical post-operative recovery and survivorship phase (3, 4). Radical hysterectomy, while curative for the primary malignancy, does not address concurrent HPV-driven benign lesions, thereby creating a distinct clinical gap in the comprehensive care of this patient population.

In gynecologic oncology practice, the persistence or recurrence of CA after cervical cancer surgery poses a multifaceted challenge. Patients are often navigating the physical and emotional sequelae of a cancer diagnosis and major surgery, including concerns about body image, sexual function, and fear of recurrence (5). The added burden of CA—with symptoms such as pruritus, pain, and bleeding—can exacerbate distress, hinder recovery, and negatively impact adherence to follow-up protocols. Therefore, developing effective, well-tolerated strategies for CA management is an integral component of holistic patient-centered care in this setting.

Standard therapies for CA, including CO_2_ laser ablation, are effective for visible lesion removal but are limited by high recurrence rates, often attributed to untreated subclinical HPV infection (6, 7). Furthermore, these modalities may not adequately address the underlying viral load or modulate the local immune microenvironment, factors crucial for sustained clearance. This underscores the need for adjunctive treatments that can target both clinical and subclinical disease while supporting host immunity.

Photodynamic therapy (PDT) has emerged as a promising modality with direct cytotoxic and immunomodulatory effects (8–10). Its application in gynecologic fields, including the treatment of vulvar intraepithelial neoplasia and CA, has shown potential for reducing viral load and inducing a favorable immune response (11, 12). PDT is a typical example of photomedicine, employing photo-activated drugs to evoke direct and indirect effects (13). In addition to photodestructive effects, PDT has recently attracted attention for photobiomodulatory effects such as immune modulation (14). These include activation of antigen-presenting cells, recruitment of immune effector cells, and modulation of cytokine profiles, specifically favoring a T helper 1 (Th1)-type immune response. These effects are particularly important in diseases associated with human papillomavirus (HPV), which are reliant on strong cell-mediated immunity for viral clearance. Hence, incorporating PDT into the therapeutic armamentarium of condyloma acuminatum may offer advantages beyond simply destroying the tissue, as is the case with many principles in oncologic photomedicine (10, 15, 16). We hypothesize that combining PDT with CO_2_ laser could synergistically overcome the limitations of laser monotherapy by providing deeper tissue clearance of HPV and enhancing local antiviral immunity.

Despite the availability of CO_2_ laser therapy and emerging evidence supporting photodynamic therapy in HPV-related conditions, there remains a lack of focused research evaluating their combined use in patients with early-stage cervical cancer complicated by condyloma acuminatum after radical surgery. In particular, limited data exist regarding their comparative effectiveness in improving not only lesion clearance but also patient-centered outcomes such as quality of life, sexual function, and immune response. Addressing this gap is essential for optimizing post-treatment survivorship care in gynecologic oncology. The primary aim of this study was to compare the effectiveness of combined photodynamic therapy and CO_2_ laser treatment versus CO_2_ laser monotherapy in relieving condyloma acuminatum symptoms in patients with early-stage cervical cancer after radical surgery. Secondary aims included evaluating differences in recurrence rates, quality of life (DLQI, FSFI, and EORTC QLQ-C30), HPV viral load reduction, cytokine profile changes, and treatment-related adverse events.

## 2 Materials and methods

### 2.1 Screening criteria

A retrospective analysis was conducted on 148 patients with early-stage cervical cancer complicated by CA who underwent radical surgery in the Department of Gynecology at our hospital between March 2022 and February 2025. The inclusion criteria were as follows: ① Preliminary diagnosis through preoperative cervical biopsy pathology, with definitive diagnosis confirmed by postoperative specimen pathology as cervical cancer (17); ② According to the International Federation of Gynecology and Obstetrics (FIGO) (18) staging, patients were classified as stage I, specifically including stages IA1 (with lymphovascular space invasion) to IB1; ③ Patients had CA located on the external genitalia or perianal skin (19), with individual wart maximum diameter ≤1 cm and number of warts ≤10; ④ Female patients aged between 25 and 60 years, with no desire for fertility; ⑤ First-time recipients of radical surgery for cervical cancer and targeted treatment for genital warts; ⑥ Underwent radical hysterectomy combined with pelvic lymphadenectomy, with standardized surgical procedures and no serious intraoperative complications; ⑦ Complete medical records and follow-up data. Exclusion Criteria: ① Previous treatment with anti-human HPV drugs, physical therapy, or immunotherapy; ② Active sexually transmitted diseases, hematological diseases, or autoimmune diseases; ③ Other malignant tumors besides cervical cancer; ④ Dysfunction of vital organs such as the heart, liver, or kidneys; ⑤ History of mental illness or cognitive dysfunction; ⑥ Allergy to any components of the medications used in the treatment; ⑦ A history of definite hypertrophic scars or keloids; ⑧ Pregnancy or lactation.

### 2.2 Grouping method

As this was a retrospective study, patients were not randomly assigned to treatment groups. The choice of treatment (CO_2_ laser alone or combined photodynamic therapy) was based on routine clinical practice, taking into account physician recommendations, patient preferences, and individual clinical characteristics. Based on the differences in treatment protocols for CA after radical surgery for cervical cancer, the 148 patients were defined into two groups: the single laser group (n=81) and the combined PDT group (n=67). The single laser group was defined as patients who received only CO_2_ laser treatment postoperatively. In contrast, the combined PDT group was defined as patients who received both CO_2_ laser and PDT treatment postoperatively.

This study is a retrospective clinical study, and its design and implementation strictly adhered to the Declaration of Helsinki (revised in 2013) and relevant ethical guidelines for medical research in China. The research protocol has been reviewed and approved by The Ethic Committee of Yantai Yuhuangding Hospital (Ethic Approval Number: K2026-212). Given that the study data were derived from clinical records collected during routine care processes without imposing any additional interventions on patients, and all included case data were anonymized using research identification numbers for data management and statistical analysis, patient privacy rights were adequately protected. After review by the ethics committee, it was determined that the conditions for waiver of informed consent were met, and no additional informed consent forms were required from the patients.

### 2.3 Cervical cancer radical surgery

All patients underwent standardized radical surgery as the primary treatment for early-stage cervical cancer. The surgical procedure consisted of a radical hysterectomy (Piver-Rutledge type III) with bilateral pelvic lymphadenectomy. The specific surgical approach (open or no-touch laparoscopic) was selected based on the surgeon’s expertise and the patient’s individual anatomical considerations, in accordance with contemporary gynecologic oncology guidelines (17). The surgical resection aimed to achieve complete tumor removal with adequate margins. This included the en bloc removal of the uterus, cervix, parametrial tissues, and the upper portion of the vagina (2–3 cm). Bilateral pelvic lymphadenectomy involved the systematic dissection of lymph node groups, including the external iliac, internal iliac, obturator, and common iliac nodes, sent for histopathological examination to determine nodal status. The same experienced gynecologic oncologist performed all surgeries. Intraoperative and immediate postoperative management followed standard protocols to minimize complications.

### 2.4 Condyloma acuminatum treatment

#### 2.4.1 Treatment timing

All patients initiated targeted treatment for genital warts 4 to 6 weeks after radical surgery for cervical cancer. Before treatment, a rigorous pre-treatment evaluation was required: a gynecological examination was conducted to confirm complete healing of the vaginal stump and abdominal incision without any signs of exudate or discharge; blood tests and inflammatory markers were assessed to rule out infection; a pelvic ultrasound was performed to exclude postoperative complications such as lymphoceles, ensuring that patients had the physical condition necessary to tolerate the treatment.

#### 2.4.2 Treatment method

1. CO_2_ Laser Treatment: Before treatment, lidocaine hydrochloride injection (Approval No. H3202342, Chengdu Better Pharmaceutical Co., Ltd., Sichuan Province) was used for local infiltration anesthesia of the lesion area. A CO_2_ laser therapy machine (Registration No. 20173014197, Wuhan Kinglight Electronics Co., Ltd., Hubei Province) was utilized, with a wavelength set at 10.6 µm and an output power of 22.4 W ± 10%. Before treatment, a 5% acetic acid test was performed to clearly identify the boundaries of the warts and any potential subclinical infection areas. Under colposcopic guidance, precise coagulation and vaporization treatments were then administered to visible warts and acetic acid-positive areas, with the treatment depth reaching 2 mm below the base of the warts.
2. PDT Treatment: PDT treatment was immediately administered following CO_2_ laser therapy. Before treatment, a precise clinical measuring ruler was used to determine the lesion area size to calculate the medication dosage. Then, 20% concentration solution of 5-aminolevulinic acid hydrochloride topical powder (Approval No. H20070027, Shanghai Fudan-Zhangjiang Bio-Pharmaceutical Co., Ltd., Shanghai) was prepared using sterile water for injection and evenly applied to the affected area and a 1 cm surrounding region. The treated area was then sealed with sterile plastic wrap for 3 hours. Subsequently, red light irradiation was performed using an LED therapy device (Registration No. 20132090940, Wuhan Yage Photonics Technology Co., Ltd., Hubei Province), with settings including a wavelength of 633 ± 10 nm and power density of 80 mW/cm², delivering a total light dose (fluence) of approximately 144 J/cm² over 30 minutes per treatment area. The light spot diameter fully covered the medicated area, and each treatment area was irradiated for 30 minutes. PDT treatment was administered once a week, with a 6-day interval between treatments, for a total of 4 consecutive treatments.

#### 2.4.3 Management and follow-up

During the treatment period, strict avoidance of sexual contact was mandated to prevent cross-infection. Patients were advised to keep the external genitalia and perianal areas clean and dry, wear loose, breathable cotton clothing, and follow a light diet, avoiding spicy, stimulating foods and alcohol consumption. Regular sleep patterns were recommended, and overexertion was to be avoided to enhance overall immune function. After the completion of the treatment course, patients were followed up once a month for a total of 6 months at the outpatient clinic.

### 2.5 Observation indicators

#### 2.5.1 Primary observation indicators

1. CA-Related Symptoms: The assessment of the relief of CA-related symptoms was based on the “World Health Organization Guidelines for the Management of Symptomatic Sexually Transmitted Infections (2021 Edition)” (20), conducted 6 months after treatment completion. If the wart area decreased by ≥60% compared to pre-treatment, with no recurrence during follow-up, and the affected skin and mucous membranes returned to normal, it was considered effective. If the wart area decreased by 20% to 59% compared to pre-treatment, with no new warts and significant alleviation of existing symptoms, it was considered partially effective. If the wart area decreased by <20% compared to pre-treatment, or if there was an increase in size or number of warts, or recurrence during follow-up, it was considered ineffective. The total effectiveness rate was calculated as (number of effective cases + number of partially effective cases)/total number of cases × 100%. Additionally, recurrence rates at 3 months and 6 months post-treatment were recorded. Recurrence was defined as the appearance of new warts within 2 cm of the original treatment site or surrounding areas, confirmed by acetic acid white test and HPV nucleic acid testing (19). The recurrence rate was calculated as the number of recurrence cases/total number of cases × 100%.
2. Quality of Life: The Dermatology Life Quality Index (DLQI) was used to assess patient quality of life before treatment and 6 months after treatment. The questionnaire consists of 10 questions, each scored on a four-point scale (0 = no effect, 1 = small effect, 2 = moderate effect, 3 = very large effect), with a total score range of 0 to 30. Higher scores indicate a more significant negative impact of CA on patient quality of life. The Cronbach’s α coefficient for this scale is 0.85 (21). Additionally, the Female Sexual Function Index (FSFI) was used to assess the sexual function of the patients. The questionnaire consists of 19 items, with each item scored on a scale from 0 to 5, resulting in a total score range of 2 to 36. Higher scores indicate better sexual function. The Cronbach’s α coefficient for this scale is 0.94 (22). To comprehensively assess the health-related quality of life (HRQOL) of patients after radical cervical cancer surgery, the European Organization for Research and Treatment of Cancer Quality of Life Questionnaire-Core 30 (EORTC QLQ-C30) was used at both pre-treatment and 6 months post-treatment. This questionnaire consists of 30 items covering five functional scales (physical, role, cognitive, emotional, and social functioning), three symptom scales (fatigue, pain, nausea and vomiting), six single-item symptoms (such as dyspnea, insomnia, loss of appetite, constipation, diarrhea, and financial difficulties), and one global health status/quality of life scale. All items are rated on a 4-point Likert scale (1 = “not at all” to 4 = “very much”), while the global health status/QOL scale is rated on a 7-point scale (1 = “very poor” to 7 = “excellent”). According to the EORTC scoring manual, scores for each dimension are linearly transformed and standardized to a range of 0-100. Higher scores in the functional scales and global health status indicate better functioning and quality of life, whereas higher scores in the symptom scales indicate greater symptom burden. The Cronbach’s α coefficient for this questionnaire ranges from 0.67 to 0.85 (23).

#### 2.5.2 Secondary observation indicators

1. Viral Load: Skin and mucosal tissue samples (sampling depth 0.3~0.5 mm) from the lesion areas were collected before treatment and after 4 weeks of treatment. The HPV DNA viral load was measured using a real-time fluorescent quantitative PCR instrument (ABI 7500, Applied Biosystems, USA). Results were expressed as ×10^6^ copies/mL.
2. Cytokines: Peripheral venous blood samples (5 mL) were collected from patients before treatment and after 4 weeks of treatment. Serum was separated by centrifugation (3000 rpm for 10 minutes). Levels of tumor necrosis factor-alpha (TNF-α), interferon-gamma (IFN-γ), interleukin-2 (IL-2), interleukin-4 (IL-4), and interleukin-10 (IL-10) were measured using an enzyme immunoassay analyzer (AXSYM, Abbott, USA) and corresponding ELISA kits (catalog numbers: CB11762, CB10293, CB10349, CB13566, Shanghai Kebio Biotechnology Co., Ltd.).
3. Adverse Reactions: Adverse reactions during the treatment period were recorded, primarily including pain, erythema/edema, and scarring.

### 2.6 Statistical analysis

Statistical analysis was performed using SPSS software (Version 29.0; developed by SPSS Inc., Chicago, IL, USA). A two-tailed p-value less than 0.05 was considered statistically significant. Based on normality assessed by the Shapiro-Wilk test, all continuous variables in this study followed a normal distribution and were reported as means ± standard deviations (M ± SD). Independent samples t-tests were used for comparisons between groups. Categorical variables were expressed as frequencies and percentages [n (%)] and compared between groups using the χ² test. Due to the retrospective nature of the study and the relatively limited sample size, multivariable regression analyses were not performed to avoid potential model overfitting.

## 3 Results

### 3.1 General information

As shown in **Table 1**, the baseline demographic and clinical characteristics were well-balanced between the two treatment groups. There were no significant differences in age (P = 0.286), BMI (P = 0.542), menopausal status (P = 0.721), surgical approach (P = 0.977), cervical cancer pathological type (P = 0.958), FIGO stage (P = 0.963), high-risk HPV type distribution (P = 0.944), CA lesion location (P = 0.970), number of warts (P = 0.126), maximum wart diameter (P = 0.140), or low-risk HPV type distribution (P = 0.939).

**Table 1:** Comparison of general information between the two groups.

Parameter	Single laser group (n=81)	Combined PDT group (n=67)	t/χ2	P
Demographics				
Age (years)	41.74 ± 7.26	40.39 ± 8.05	1.072	0.286
BMI (kg/m2)	22.58 ± 2.43	22.32 ± 2.61	0.611	0.542
Menopause [n(%)]	35 (43.21%)	27 (40.30%)	0.128	0.721
Cervical cancer-related				
Surgical approach [n(%)]			0.001	0.977
Laparoscopic	24 (29.63%)	20 (29.85%)		
Open	57 (70.37%)	47 (70.15%)		
Pathological type [n(%)]			0.086	0.958
Squamous cell carcinoma	57 (70.37%)	48 (71.64%)		
Adenocarcinoma	16 (19.75%)	12 (17.91%)		
Adenosquamous carcinoma	8 (9.88%)	7 (10.45%)		
FIGO staging [n(%)]			0.075	0.963
IA1	12 (14.81%)	11 (16.42%)		
IA2	28 (34.57%)	23 (34.33%)		
IB1	41 (50.62%)	33 (49.25%)		
High risk HPV typing [n(%)]			0.115	0.944
16	45 (55.56%)	38 (56.72%)		
18	16 (19.75%)	14 (20.90%)		
Others^a^	20 (24.69%)	15 (22.39%)		
CA-related				
Wart location [n(%)]			0.061	0.970
External genitalia	53 (65.43%)	45 (67.16%)		
Perianal skin	22 (27.16%)	17 (25.37%)		
Mixed	6 (7.41%)	5 (7.46%)		
Number of warts	6.12 ± 1.54	5.69 ± 1.81	1.540	0.126
Individual wart maximum diameter (cm)	0.62 ± 0.16	0.58 ± 0.18	1.484	0.140
Low risk HPV typing [n(%)]			0.126	0.939
6	56 (69.14%)	48 (71.64%)		
11	18 (22.22%)	14 (20.90%)		
Others^a^	7 (8.64%)	5 (7.46%)		

PDT, Photodynamic Therapy; BMI, Body Mass Index; FIGO, International Federation of Gynecology and Obstetrics; CA, Condyloma Acuminatum; HPV, Human Papillomavirus; ^a^indicates other types or not detected.

### 3.2 Condyloma acuminatum-related symptoms

The combined PDT group demonstrated a significantly higher overall effectiveness rate in relieving CA-related symptoms compared to the single laser group (94.03% vs. 77.78%, P = 0.006; **Table 2**).

**Table 2:** Comparison of the relief effect of CA-related symptoms between the two groups [n(%)].

Parameter	Single laser group (n=81)	Combined PDT group (n=67)	χ2	P
Overall effectiveness rate	63 (77.78%)	63 (94.03%)	7.653	0.006
Effective	24 (29.63%)	27 (40.30%)		
Partially effective	39 (48.15%)	36 (53.73%)		
Ineffective	18 (22.22%)	4 (5.97%)		

CA, Condyloma Acuminatum; PDT, Photodynamic Therapy.

Recurrence rates at both the 3-month and 6-month follow-ups were significantly lower in the combined PDT group than in the single laser group (7.46% vs. 24.69%, P = 0.005; and 11.94% vs. 34.57%, P = 0.001, respectively; **Table 3**).

**Table 3:** Comparison of the recurrence rates of CA between two groups [n(%)].

Parameter	Single laser group (n=81)	Combined PDT group (n=67)	χ2	P
3 months after treatment	20 (24.69%)	5 (7.46%)	7.753	0.005
6 months after treatment	28 (34.57%)	8 (11.94%)	10.199	0.001

CA, Condyloma Acuminatum; PDT, Photodynamic Therapy.

### 3.3 Quality of life

**Figure 1:**
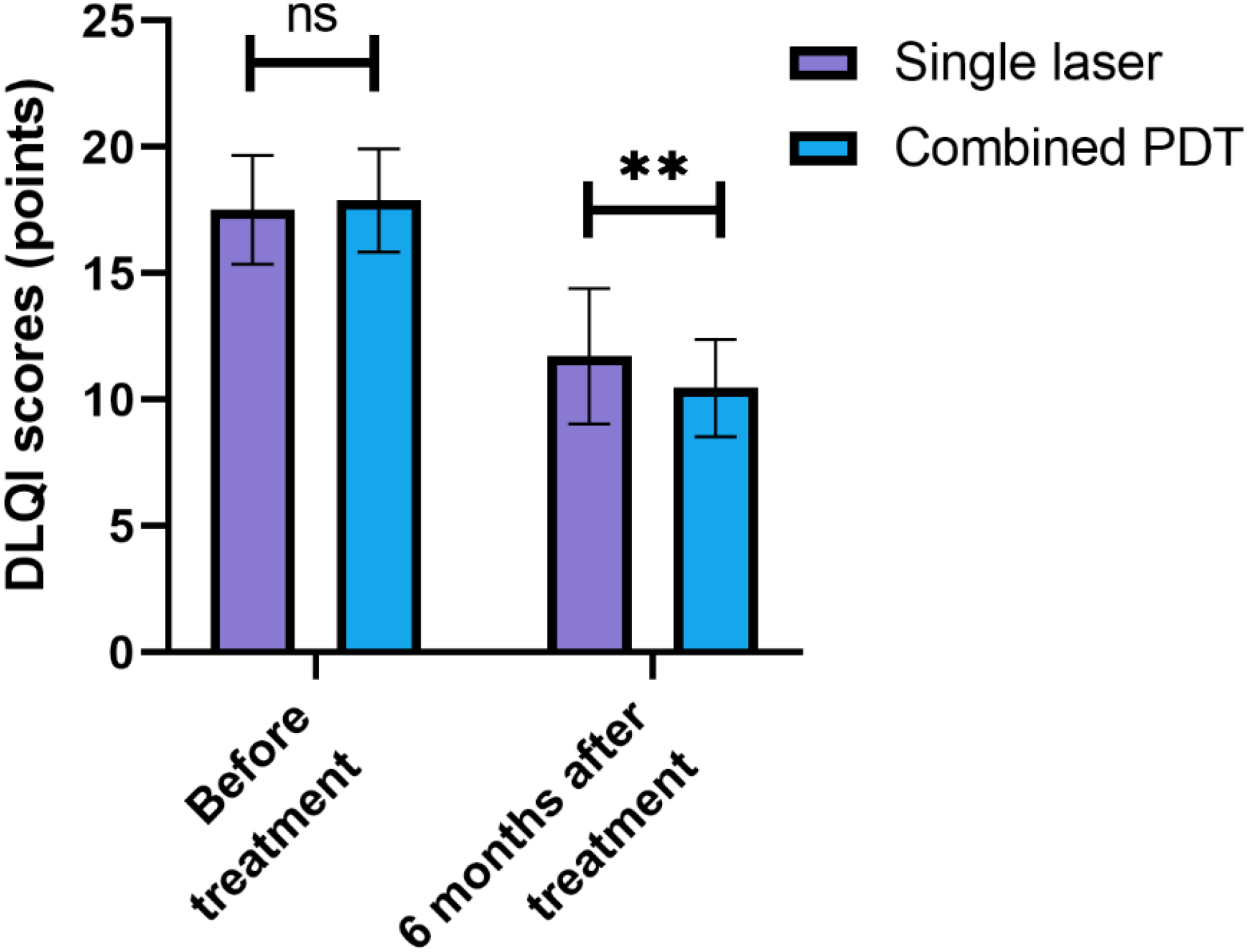
Comparison of the DLQI scores between two groups (points). The DLQI score was significantly lower in the combined group compared to the control group, indicating a meaningful improvement in patients’ dermatology-related quality of life. DLQI, Dermatology Life Quality Index; PDT, Photodynamic Therapy; ns, no significant difference; **:P<0.01.

Both groups showed a reduction in DLQI scores from baseline to 6 months post-treatment, indicating improved dermatology-specific quality of life (**Figure 1**). While the pre-treatment scores were comparable between groups (P = 0.283), the post-treatment score in the combined PDT group (10.46 ± 1.92) was significantly lower than that in the single laser group (11.73 ± 2.68), demonstrating a superior improvement in the combined PDT group (t=3.361, P = 0.001).

**Figure 2:**
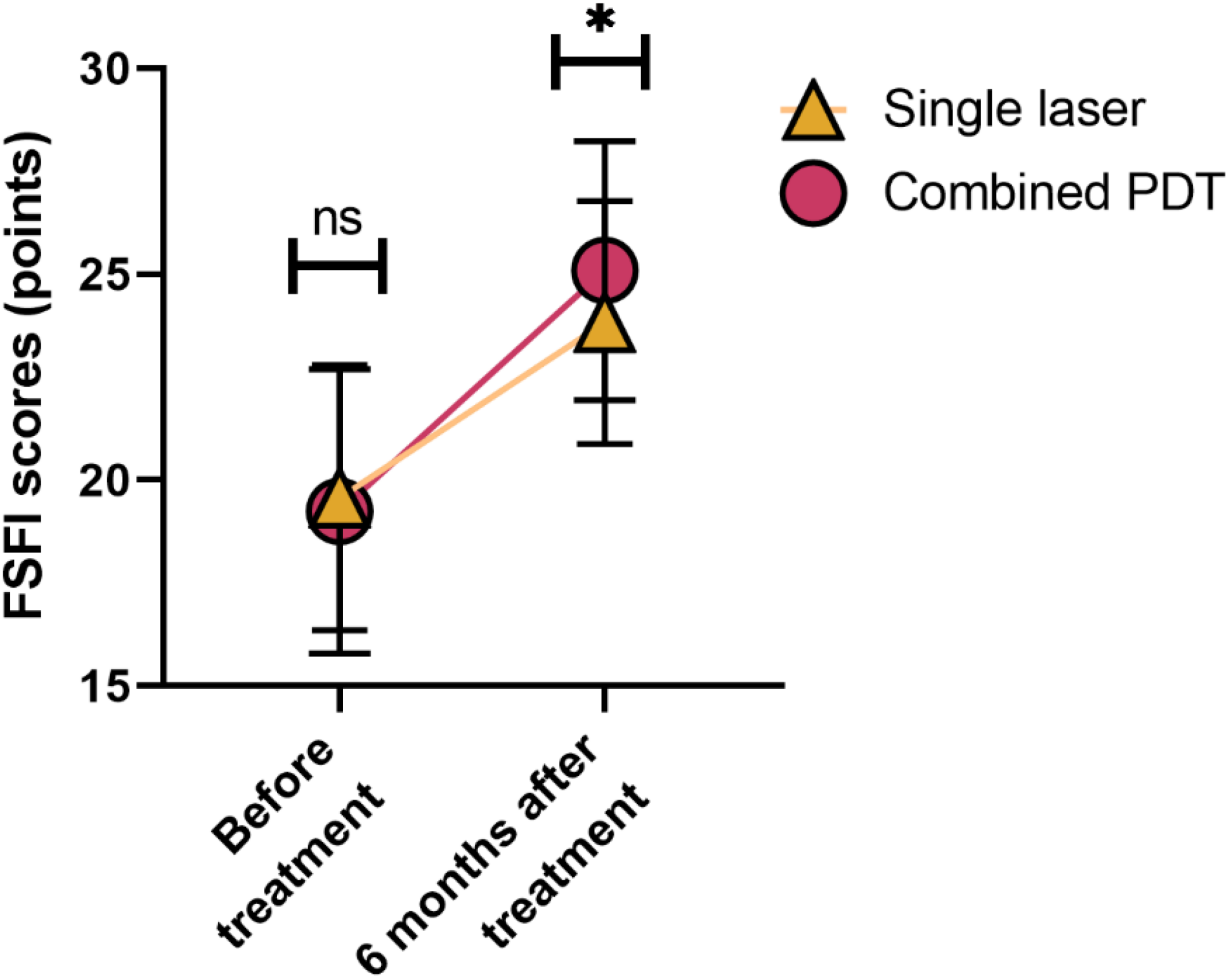
Comparison of the FSFI scores between two groups (points). FSFI, Female Sexual Function Index; PDT, Photodynamic Therapy; ns, no significant difference; *:P<0.05.

At baseline, FSFI scores were comparable between the groups (P = 0.551) (**Figure 2**). However, at the 6-month assessment, the combined PDT group achieved a significantly higher mean FSFI score (25.09 ± 3.14) compared to the single laser group (23.83 ± 2.95) (t=2.515, P = 0.013).

The HRQOL of early cervical cancer patients was further evaluated using the EORTC QLQ-C30 questionnaire. As presented in **Table 4**, there were no significant differences in any of the functional scales, symptom scales, or global health status between the two groups at baseline (all P > 0.05). At the 6-month follow-up, the combined PDT group demonstrated significantly better outcomes across multiple dimensions compared to the single laser group. In terms of functional scales, the combined PDT group exhibited significantly higher scores in physical functioning (P = 0.003), role functioning (P = 0.008), cognitive functioning (P = 0.032), emotional functioning (P = 0.039), and social functioning (P = 0.047), indicating superior functional recovery and psychosocial adaptation. Regarding symptom burden, the combined PDT group showed significantly lower scores in fatigue (P = 0.008), pain (P = 0.009), nausea and vomiting (P = 0.023), dyspnea (P = 0.011), insomnia (P = 0.007), appetite loss (P = 0.011), constipation (P = 0.041), diarrhea (P = 0.031), and financial difficulties (P = 0.026), reflecting a lower symptom burden and better overall comfort. Finally, the global health status/quality of life score was significantly higher in the combined PDT group (P = 0.009), suggesting a more favorable overall HRQOL outcome compared to the single laser group.

**Table 4:** Comparison of the EORTC QLQ-C30 scores between the two groups (points).

Parameter	Single laser group (n=81)	Combined PDT group (n=67)	t	P
Physical functioning				
Before treatment	78.45 ± 8.32	79.12 ± 7.95	0.494	0.622
6 months after treatment	82.16 ± 7.84	85.73 ± 6.52	2.969	0.003
Role functioning				
Before treatment	75.62 ± 9.14	76.08 ± 8.67	0.315	0.754
6 months after treatment	79.45 ± 8.23	82.91 ± 7.15	2.705	0.008
Cognitive functioning				
Before treatment	80.34 ± 7.55	81.02 ± 6.88	0.567	0.572
6 months after treatment	83.27 ± 6.92	85.56 ± 5.74	2.163	0.032
Emotional functioning				
Before treatment	72.18 ± 10.24	71.85 ± 9.67	0.205	0.838
6 months after treatment	76.52 ± 9.35	79.47 ± 7.48	2.087	0.039
Social functioning				
Before treatment	74.56 ± 8.91	75.23 ± 8.42	0.470	0.639
6 months after treatment	78.34 ± 7.63	80.76 ± 6.85	2.003	0.047
Fatigue				
Before treatment	38.45 ± 9.67	37.92 ± 8.54	0.350	0.727
6 months after treatment	32.18 ± 8.23	28.73 ± 7.16	2.690	0.008
Pain				
Before treatment	24.56 ± 6.34	25.11 ± 5.88	0.545	0.587
6 months after treatment	18.47 ± 5.52	16.21 ± 4.67	2.654	0.009
Nausea and vomiting				
Before treatment	8.45 ± 2.23	8.12 ± 2.97	0.748	0.456
6 months after treatment	7.56 ± 1.84	6.84 ± 1.95	2.294	0.023
Dyspnea				
Before treatment	12.34 ± 3.67	12.89 ± 3.12	0.977	0.330
6 months after treatment	10.44 ± 2.92	9.23 ± 2.78	2.562	0.011
Insomnia				
Before treatment	30.41 ± 8.36	29.87 ± 7.96	0.401	0.689
6 months after treatment	25.67 ± 7.52	22.54 ± 6.23	2.723	0.007
Appetite loss				
Before treatment	15.23 ± 4.78	14.89 ± 4.14	0.454	0.650
6 months after treatment	12.41 ± 3.66	11.15 ± 2.23	2.575	0.011
Constipation				
Before treatment	18.34 ± 5.12	17.92 ± 4.78	0.520	0.604
6 months after treatment	15.23 ± 4.46	13.94 ± 3.16	2.058	0.041
Diarrhea				
Before treatment	6.78 ± 2.05	6.48 ± 2.11	0.854	0.394
6 months after treatment	6.12 ± 1.97	5.53 ± 1.34	2.178	0.031
Financial difficulties				
Before treatment	22.43 ± 6.89	21.98 ± 6.36	0.416	0.678
6 months after treatment	20.12 ± 5.78	18.15 ± 4.67	2.248	0.026
Global health status/quality of life				
Before treatment	65.37 ± 9.12	66.01 ± 8.67	0.435	0.664
6 months after treatment	72.49 ± 8.23	75.87 ± 7.14	2.633	0.009

EORTC QLQ-C30, European Organization for Research and Treatment of Cancer Quality of Life Questionnaire - Core 30; PDT, Photodynamic Therapy.

### 3.4 Viral load

The pre-treatment viral loads were similar (P = 0.669), suggesting improved viral clearance and potential reduction in recurrence risk (**Table 5**). Following treatment, the combined PDT group exhibited a dramatically lower mean viral load (0.97 ± 0.15) compared to the single laser group (2.44 ± 0.31), a difference that was highly statistically significant (t=37.518, P<0.001).

**Table 5:** Comparison of the HPV load between two groups (×10^6^ copies/mL).

Parameter	Single laser group (n=81)	Combined PDT group (n=67)	t	P
Before treatment	5.48 ± 0.62	5.52 ± 0.59	0.428	0.669
After 4 weeks of treatment	2.44 ± 0.31	0.97 ± 0.15	37.518	< 0.001

HPV, Human Papillomavirus; PDT, Photodynamic Therapy.

### 3.5 Cytokines

The cytokine profiles before and after treatment are detailed in **Table 6**. Baseline levels of TNF-α, IFN-γ, IL-2, IL-4, and IL-10 did not differ significantly between the groups (all P>0.05). After 4 weeks of treatment, the combined PDT group showed a substantially greater increase in IFN-γ levels (8.32 ± 1.43 pg/mL vs. 3.54 ± 1.05 pg/mL, P<0.001) and IL-2 levels (39.25 ± 6.36 pg/mL vs. 37.18 ± 5.92 pg/mL, P = 0.043), alongside a more pronounced decrease in IL-4 (4.05 ± 0.96 pg/mL vs. 4.53 ± 1.34 pg/mL, P = 0.012) and IL-10 (5.52 ± 1.03 pg/mL vs. 5.97 ± 1.44 pg/mL, P = 0.028) compared to the single laser group. A smaller but significant increase in TNF-α was also noted in the combined PDT group (31.76 ± 5.12 pg/mL vs. 29.34 ± 4.83 pg/mL, P = 0.004).

**Table 6:** Comparison of the cytokine levels between the two groups (pg/mL).

Parameter	Single laser group (n=81)	Combined PDT group (n=67)	t	P
TNF-α				
Before treatment	28.56 ± 5.42	29.12 ± 5.67	0.613	0.541
After 4 weeks of treatment	29.34 ± 4.83	31.76 ± 5.12	2.953	0.004
IFN-γ				
Before treatment	3.25 ± 0.87	3.18 ± 0.91	0.479	0.632
After 4 weeks of treatment	3.54 ± 1.05	8.32 ± 1.43	22.733	< 0.001
IL-2				
Before treatment	36.45 ± 6.84	35.92 ± 7.09	0.463	0.644
After 4 weeks of treatment	37.18 ± 5.92	39.25 ± 6.36	2.041	0.043
IL-4				
Before treatment	4.82 ± 1.23	4.76 ± 1.18	0.320	0.750
After 4 weeks of treatment	4.53 ± 1.34	4.05 ± 0.96	2.533	0.012
IL-10				
Before treatment	6.35 ± 1.62	6.28 ± 1.57	0.239	0.812
After 4 weeks of treatment	5.97 ± 1.44	5.52 ± 1.03	2.214	0.028

PDT, Photodynamic Therapy; TNF-α, Tumor Necrosis Factor-alpha; IFN-γ, Interferon-gamma; IL-2, Interleukin-2; IL-4, Interleukin-4; IL-10, Interleukin-10.

### 3.6 Adverse reactions

The incidence of adverse reactions differed between the groups (**Table 7**). Erythema/edema was more frequently reported in the combined PDT group (43.28% vs. 19.75%, P = 0.002), whereas scarring occurred less often (4.48% vs. 16.05%, P = 0.024). The reported rates of pain did not differ significantly (P = 0.102).

**Table 7:** Comparison of the incidence of adverse reactions between two groups [n(%)].

Parameter	Single laser group (n=81)	Combined PDT group (n=67)	χ^2^	P
Pain	35 (43.21%)	38 (56.72%)	2.676	0.102
Erythema/Edema	16 (19.75%)	29 (43.28%)	9.595	0.002
Scarring	13 (16.05%)	3 (4.48%)	5.092	0.024

PDT, Photodynamic Therapy.

## 4 Discussion

The management of CA in patients with early-stage cervical cancer presents unique challenges, particularly after radical surgery. This study aimed to evaluate the impact of PDT combined with CO_2_ laser treatment compared to CO_2_ laser alone on symptom relief, quality of life, viral load reduction, and cytokine profiles in these patients. The findings suggest that the combination of PDT and CO_2_ laser may offer superior outcomes in several key areas.

These findings are consistent with previous studies reporting that CO_2_ laser therapy is effective for lesion removal but associated with relatively high recurrence rates. In contrast, photodynamic therapy has demonstrated benefits in reducing HPV viral load and enhancing local immune responses (24, 25). Photodynamic therapy involves the use of a photosensitizer activated by light, leading to the production of reactive oxygen species that damage both viral particles and infected cells (1, 26). This direct cytotoxic effect may contribute to more thorough eradication of CA lesions, thereby reducing symptoms such as itching, pain, and bleeding. PDT can induce an immune response that targets residual or recurring viral infections, further enhancing its therapeutic efficacy (27). From a gynecologic oncology perspective, effective control of these bothersome CA symptoms is crucial, as they can compound the physical discomfort and psychological distress already present following major cancer surgery, potentially impacting recovery and adherence to follow-up care.

Recurrence rates at both 3-month and 6-month follow-ups were lower in the combined PDT group compared to the single laser group. The prolonged remission observed in the PDT group might be due to the sustained immune activation induced by PDT. By stimulating the release of cytokines and recruiting immune cells to the site of infection, PDT enhances the body’s ability to clear the virus and prevent recurrence. CO_2_ laser treatment primarily relies on physical ablation of visible lesions, which may leave behind subclinical infections that can lead to higher recurrence rates (28–30). For the gynecologist managing a cervical cancer survivor, minimizing CA recurrence is paramount. Frequent recurrences necessitate repeated clinic visits, cause ongoing patient anxiety, and, in the context of a history of HPV-driven malignancy, may contribute to patient concerns about cancer recurrence, thereby affecting long-term psychosocial well-being.

Both groups showed improvements in dermatology-specific quality of life as measured by the DLQI. However, the combined PDT group demonstrated greater improvements. This enhanced quality of life could be linked to better symptom control and reduced recurrence rates, allowing patients to experience fewer disruptions in their daily activities. Furthermore, the combined PDT approach may have a positive impact on sexual health, as evidenced by the higher FSFI scores in this group. Improved sexual function could result from less frequent CA recurrences and better psychological well-being, contributing to overall quality of life (8, 31, 32). Sexual dysfunction is a well-documented sequelae of cervical cancer (33). Therefore, a treatment that mitigates CA-related sexual morbidity represents a valuable adjunct in the comprehensive care of these patients.

The assessment of health-related quality of life using the EORTC QLQ-C30 provides a comprehensive, cancer-specific perspective highly relevant to gynecologic oncology practice. Our results demonstrate that the combined PDT group achieved significantly greater improvements not only in dermatology-specific (DLQI) and sexual health (FSFI) metrics but also across core functional and symptom domains pertinent to cancer survivors. The superior scores in physical, role, emotional, and social functioning suggest that the combined therapy contributes to a broader and more robust recovery after radical surgery. The significant reduction in symptom burden—particularly in fatigue, pain, and insomnia—is critically important, as these are common and debilitating issues following cervical cancer treatment (5). The higher global health status score further underscores that integrating PDT may offer a holistic benefit, enhancing the patient’s overall sense of well-being during a vulnerable post-operative period. This aligns with the growing emphasis in gynecologic oncology on optimizing not just survival but also patient-reported outcomes and quality of survival.

Reduction in viral load was more pronounced in the combined PDT group compared to the single laser group. This finding suggests that PDT not only eliminates visible CA lesions but also effectively reduces the viral burden within the tissue. The mechanism underlying this effect likely involves the destruction of infected cells and inhibition of viral replication through oxidative stress. PDT-induced cell death triggers an inflammatory response that helps eliminate residual viral particles and prevents new infections from establishing (34, 35). CO_2_ laser treatment may not address the underlying viral reservoirs as effectively, leading to higher viral loads post-treatment. From a gynecologic standpoint, reducing the HPV viral load in the anogenital field is desirable, as it may theoretically lower the risk of recurrent or metachronous HPV-related dysplasia, even if the dominant oncogenic types differ from the low-risk types causing CA.

Changes in cytokine profiles provide insights into the immunological effects of the treatments. The combined PDT group exhibited a greater increase in IFN-γ and IL-2, alongside a more pronounced decrease in IL-4 and IL-10. These changes indicate a shift towards a Th1-dominant immune response, which is crucial for effective antiviral immunity. IFN-γ and IL-2 play essential roles in activating macrophages, natural killer cells, and T lymphocytes, enhancing their ability to combat viral infections. The reduction in IL-4 and IL-10 levels suggests a suppression of the Th2 immune response, which is typically associated with chronic infections and inflammation (36, 37). This modulation of the immune response may contribute to the improved clinical outcomes observed in the combined PDT group (38). Promoting a robust Th1 response is particularly interesting in the context of HPV-associated disease, as cell-mediated immunity is key to viral clearance (39). An adjunctive therapy that non-specifically enhances antiviral immunity could be beneficial for patients who have undergone cancer surgery and may face immunologic challenges. From a mechanistic photomedicine perspective, 5-aminolevulinic acid-mediated PDT at 633 nm may initiate a cascade of immunological events beyond localized cytotoxicity (40). Upon activation, PDT induces immunogenic cell death, leading to the release of damage-associated molecular patterns (DAMPs), which facilitate dendritic cell maturation and antigen presentation (41). This process enhances adaptive immune activation, particularly promoting a Th1-skewed response characterized by increased IFN-γ and IL-2 production. These cytokines play critical roles in activating cytotoxic T lymphocytes and natural killer cells, which are essential for clearing HPV-infected cells (42). Concurrently, the observed reduction in IL-4 and IL-10 suggests suppression of Th2-mediated immunosuppressive pathways. This shift toward a Th1-dominant immune profile provides a plausible explanation for the reduced recurrence rates and improved clinical outcomes observed in the combined PDT group (43, 44). These findings further position PDT within the broader framework of photomedicine, where light-based therapies exert both direct and immune-mediated therapeutic effects (45).

While both treatments were generally well-tolerated, there were differences in adverse reactions between the groups. Erythema and edema were more frequently reported in the combined PDT group, likely due to the inflammatory response induced by PDT. However, scarring occurred less often in this group, suggesting that the combined approach may result in fewer long-term complications. Pain levels did not differ significantly between the groups, indicating that the discomfort associated with each treatment modality is comparable. Understanding these side effects is crucial for optimizing patient care and ensuring informed decision-making regarding treatment options (46, 47). In gynecologic practice, minimizing scarring in the vulvoperineal area is important for both aesthetic and functional reasons, including future sexual comfort. From a clinical perspective, these findings have important implications for gynecologic oncology survivorship care. Patients recovering from cervical cancer treatment often experience persistent HPV infection, recurrence of condyloma acuminatum, and reduced quality of life. The potential benefits of combined photodynamic therapy and CO_2_ laser treatment in improving symptom control, reducing recurrence risk, and enhancing quality of life suggest that this approach may represent a valuable addition to post-treatment management strategies. However, careful patient selection and further prospective validation are required.

This study has some limitations. First, this study is retrospective, which introduces biases, including selection bias and non-random allocation of treatment. The choice of combined PDT treatment or CO_2_ laser ablation was determined by clinical discretion, patient choice, lesion characteristics, and the physician’s experience, rather than randomization. This could have led to potential confounders affecting treatment efficacy. While the baseline characteristics of the two groups were similar, other factors, such as immune status, lesion biology, or socioeconomic factors, may have influenced the results. Prospective randomized controlled trials (RCTs) with uniform treatment allocation are needed to confirm these results and avoid potential bias. These should also include longer follow-ups to assess long-term outcomes and recurrence rates. Furthermore, the short-term follow-up (six months) hampers the evaluation of long-term efficacy, and the single-center design of this study may limit its generalizability to other populations. In addition, effect sizes and confidence intervals were not calculated, which may limit the quantitative interpretation of the magnitude and precision of the observed differences.

The integration of PDT with CO_2_ laser treatment offers potential advantages in managing CA in patients with early-stage cervical cancer. This combined approach appears to enhance symptom relief, reduce recurrence rates, improve quality of life, and decrease viral loads. Further prospective studies are needed to validate these findings and to establish the optimal application of this combined approach in clinical practice. Enhanced understanding and application of these therapeutic strategies will contribute to improved management of CA and better holistic patient outcomes in gynecologic oncology.

## 5 Conclusion

Combined photodynamic therapy and CO_2_ laser treatment was associated with improved clinical outcomes and quality of life compared to CO_2_ laser alone in patients with condyloma acuminatum after cervical cancer surgery. However, these findings should be interpreted with caution due to the retrospective design of the study. The integrated approach appears to enhance symptom relief and improve quality of life, possibly through more effective reduction of viral load and modulation of immune responses. Patients receiving combined PDT showed a trend towards lower recurrence rates and better sexual health outcomes. However, further prospective, well-designed studies are needed to confirm these findings and establish causal relationships.

## Data Availability

The original contributions presented in the study are included in the article/supplementary material. Further inquiries can be directed to the corresponding authors.
